# Incremental effect and risk association of obstructive coronary artery disease on microvascular myocardial ischemia in T2DM patients evaluated via CT myocardial perfusion

**DOI:** 10.3389/fcvm.2026.1897324

**Published:** 2026-09-22

**Authors:** Wangshu Zhu, Yijia Xiong, Yidong Jin, Runjianya Ling, Geng Gu, Zhihan Xu, Jian Ma, Yue-Hua Li

**Affiliations:** 1 Department of Diagnostic and Interventional Radiology, Shanghai Sixth People's Hospital Affiliated to Shanghai Jiao Tong University School of Medicine, Shanghai, China; 2 Siemens Healthineers China, Shanghai, China; 3 Department of Cardiology, Shanghai Sixth People's Hospital Affiliated to Shanghai Jiao Tong University School of Medicine, Shanghai, China

**Keywords:** CT myocardial perfusion, microvascular myocardial ischemia, obstructive coronary artery disease, prognosis, type 2 diabetes mellitus

## Abstract

**Objective:**

Microvascular myocardial ischemia (MMI) in patients with T2DM may be influenced by the presence of concomitant obstructive coronary artery disease (OCAD). Our investigation focused on assessing the impact of OCAD on MMI and delineating the prognostic relevance of MMI.

**Methods:**

This study included four groups: (1) control group without OCAD (*n* = 125), (2) control group with OCAD (*n* = 99); (3) T2DM patients without OCAD (*n* = 100); (4) T2DM patients with OCAD (*n* = 93). All patients received dynamic computed tomography myocardial perfusion imaging (CT–MPI) as well as coronary computed tomography angiography (CCTA). Univariate and multivariate linear regression analyses were performed to identify independent determinants of clinical characteristics and perfusion metrics related to MMI. Major adverse cardiovascular events (MACEs) were documented, and the prognostic significance of CT–MPI compared with clinical variables was assessed.

**Results:**

The myocardial blood flow (MBF) stepwise reduced from the control (OCAD−), T2DM (OCAD−), control (OCAD+) to T2DM (OCAD+) group (*p* < 0.05). Independent associations were observed between global MBF and OCAD or T2DM (OCAD: *p* < 0.05, R^2^ = 0.154; T2DM: *β* = −11.96, *p* < 0.001, R^2^ = 0.101). A significant association was observed between OCAD and global MBF in the circumstance of T2DM (R^2^ = 0.176). An independent association was observed between the global MBF and MACEs (*p* < 0.05).

**Conclusion:**

Dynamic CT–MPI combined with CCTA demonstrated that the presence of OCAD in T2DM patients is associated with a greater occurrence of MMI. MBF emerged as a robust predictor of cardiovascular outcomes in this population.

## 1 Introduction

Among individuals with diabetes mellitus, cardiovascular disease persists as the predominant driver of morbidity and mortality and represents a major component of their cumulative health burden. In diabetic patients, cardiovascular involvement contributes to an elevated risk of adverse cardiovascular outcome (1). Previous research has shown that, compared with non-diabetic individuals, those with DM exhibit a greater burden and extent of coronary atherosclerosis, as well as a higher frequency of subclinical atherosclerotic lesions without overt is chemic manifestations (2). Furthermore, DM affects the coronary microvasculature and epicardial coronary arteries (3) and represents an independent contributor for coronary atherosclerosis and heart failure (4).

DM leads to endothelial dysfunction, which consequently causes widespread impairment of coronary microvascular function (5). A prior study demonstrated that the prevalence of microvascular myocardial ischemia (MMI) was greater in patients with DM who lacked obstructive coronary artery disease (OCAD) than in those without DM. and confirmed the association between MMI and angina (6). However, Evidence increasingly shows that MMI is associated with increased cardiovascular death and poorer clinical outcomes among individuals with T2DM and OCAD (7, 8).

Dynamic computed tomography–myocardial perfusion imaging (CT–MPI) in conjunction with coronary CT angiography (CCTA) is a “one-stop-shop” imaging protocol that provides information about coronary anatomy and myocardial perfusion (9). CCTA has been extensively utilized and serves a critical function in the accurate evaluation of obstructive stenosis within the epicardial coronary arteries (10). Dynamic CT–MPI quantifies myocardial blood flow (MBF) and has shown strong agreement with invasive fractional flow reserve for diagnostic evaluation (11). Relative to static CT–MPI, dynamic CT–MPI generates quantitative perfusion metrics that improve the accuracy of myocardial ischemia assessment (12). Nevertheless, it is still uncertain whether this approach can effectively assess the additional effect of epicardial coronary obstruction on microvascular myocardial injury in patients with T2DM.

This retrospective study sought to assess the influence of OCAD on MMI in patients with T2DM and to further delineate the association between prognosis of coronary obstruction and MMI.

## 2 Methods

### 2.1 Patient population

Ethical approval for this retrospective study was granted by the institutional ethics committee board (approval No: 2020-162). Between January 2017 and May 2023, this study included patients at our institution with clinically confirmed T2DM who underwent integrated CT-MPI and CCTA. The patients’ demographic parameters, baseline clinical risk factors, laboratory data, clinical symptom, and radiation dose were recorded from medical files and telephone follow-up. The diagnostic criteria for DM were defined according to previously published studies (13). Exclusion criteria included: 1) prior myocardial infarction or coronary revascularization, 2) hemodynamically significant lesion in the corresponding segment, 3) other cardiomyopathy, 4) and severely impaired CT image quality and missing data. Especially, the “lesions with hemodynamic significance” were defined as the patients were excluded when myocardial ischemia was identified in the myocardial territory supplied by an obstructive coronary artery segment, as this finding could be attributed to epicardial coronary artery disease rather than microcirculatory dysfunction. In contrast, if a patient with OCAD showed no ischemia in the myocardial territory supplied by the obstructive coronary artery, but ischemia was identified in a myocardial territory supplied by a non-obstructive coronary artery, the patient was considered to have MMI. MMI was characterized by imaging-confirmed ischemic changes in the absence of OCAD for the corresponding segment (14). According to previous studies, myocardial ischemia was defined as MBF critical value of 100 mL/min/100 mL (11), and 105 mL/min/100 mL was the lower limit (15). Therefore, in this study, MBF value of 100 mL/min/100 mL was used as the critical value to distinguish ischemia, and MMI was defined as more than or equal to 2 ischemic segments without obstructive stenosis in the corresponding myocardial segment (6). Patients without T2DM and OCAD, and patients with OCAD and without T2DM, who received CT–MPI and CCTA during January 2017 to May 2023 were retrospectively selected as the control cohort, following application of the exclusion criteria and matching of demographic and clinical risk profiles. The flowchart showing the participant inclusion and exclusion is presented in Figure 1**.** Finally, 417 patients, 224 in the control group [including with no OCAD (*n* = 125) and with OCAD (*n* = 99)], and 193 in T2DM group [including T2DM with no OCAD (*n* = 100) and T2DM with OCAD (*n* = 93)], were initially screened.

**Figure 1:**
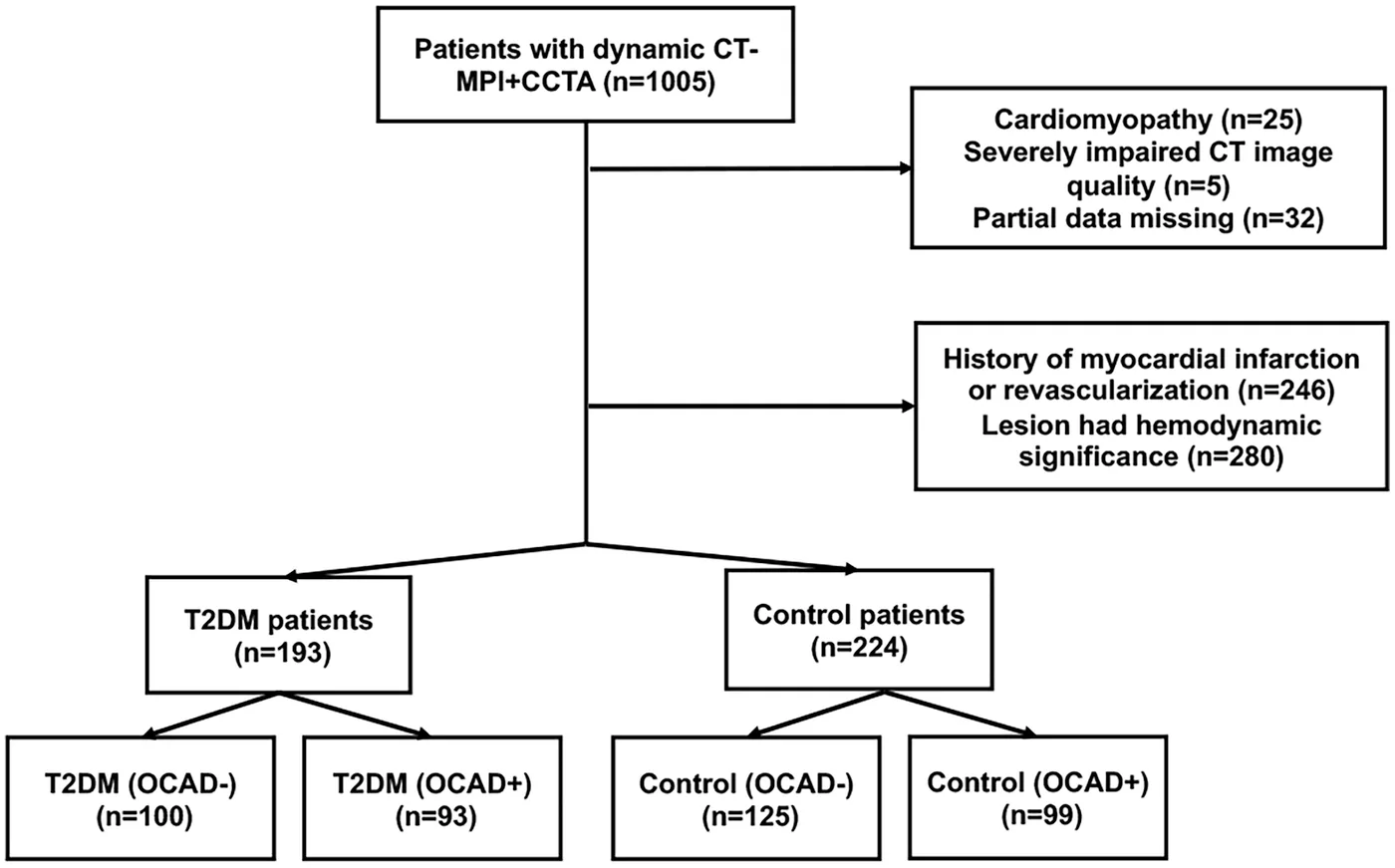
Flowchart showing study participant inclusion and exclusion.

### 2.2 Imaging protocols

CT imaging was conducted using a dual-source scanner (SOMATOM Force, Siemens), with an integrated protocol that included coronary artery calcium scoring (CACS), dynamic CT–MPI, and CCTA. CACS was performed first to assess the extent of calcification in each epicardial coronary vessel. Adenosine triphosphate was intravenously infused over 3 min at 160 µg/kg/min before triggering the MPI acquisition. Dynamic CT-MPI was then acquired in shuttle mode, initiated 4 s after contrast injection, using a fixed dose of 48 mL of iopromide. Scanning was performed in end-systole, approximately 250 ms following the R wave, with sampling intervals adjusted to one to three cardiac cycles based on heart rate. Five minutes after dynamic CT–MPI, sublingual nitroglycerin was given. Coronary CT angiography was performed with prospective ECG-triggered sequential scanning, administering 1.4 mL/kg of iopromide The full technical specifications of CT–MPI, CCTA, and contrast medium injection are presented in the Supplementary Materials.

### 2.3 CT–MPI and CCTA analysis

A dedicated commercial platform (Myocardial Perfusion Analysis, VPCT Body; Siemens Healthineers, Forchheim, Germany) was employed for quantitative perfusion analysis (16). Manual motion correction was applied when breathing-induced misregistration of the left ventricle occurred. Perfusion measurements were obtained by manually delineating regions of interest (ROI) on basal short-axis slices following the 17-segment schema, omitting the apical segment (17). Each ROI encompassed the entire myocardial segment while avoiding the epicardial and endocardial borders. Global perfusion metrics were calculated as the mean values across all 17 segments. Myocardial blood flow (MBF, mL/min/100 mL), myocardial blood volume (MBV, mL/100 mL), perfusion capillary blood volume (PCBV, mL/100 mL), flow extraction (FE, mL/min/100 mL), and time to peak (TTP, sec) were independently assessed by two cardiovascular radiologists with 5 and 10 years of experience, respectively, who were blinded to all clinical information. Perfusion analysis was conducted separately from CCTA interpretation with a minimum 2-week interval between sessions. Any discrepancies were resolved by consensus.

Coronary stenosis was assessed manually, and stenosis severity was classified using the Coronary Artery Disease–Reporting and Data System (CAD-RADS) (18). Among patients with T2DM, CAD-RADS categories 0–2 were defined as no obstructive coronary artery disease (OCAD–), whereas grades 3–5 indicated the presence of OCAD (OCAD+). All CCTA parameters were independently evaluated and Disagreements were resolved by reaching a consensus.

### 2.4 Outcomes

Data regarding clinical outcomes were retrieved from patients’ electronic health records. Major adverse cardiovascular events (MACEs) MACE was defined as all-cause mortality, cardiac death, nonfatal myocardial infarction, late revascularization, or rehospitalization due to heart failure or aggravated angina. Only the first event occurring after CT–MPI was considered. Event time was defined as the interval between the CT–MPI examination and the first recorded event. Patients without any MACEs were censored at their last available follow-up.

### 2.5 Statistical analysis

The distribution of continuous variables was evaluated using the one-sample Kolmogorov–Smirnov test. Data with normal distribution are summarized as mean ± standard deviation; skewed variables are described using the median and interquartile range. Group comparisons across the four cohorts were performed using one-way analysis of variance with Bonferroni *post-hoc* adjustment, or the Kruskal–Wallis test when the assumption of normality was not satisfied. Intra- and interobserver reproducibility for all quantitative parameters was assessed by calculating intraclass correlation coefficients. To investigate the individual and synergistic impact of OCAD and T2DM on CT–MPI–derived parameters, univariate and multivariate linear regression analyses were applied. Variables with a univariate *p* value < 0.1 were subsequently included in multivariable models using a stepwise selection procedure. Multivariable linear regression models were then constructed with sex, age, and angina as adjustment covariates: Model 1 evaluated T2DM, Model 2 evaluated CAD-RADS with CAD-RADS 0 as the reference, and Model 3 included both T2DM and CAD-RADS. Time-to-event data were evaluated using Kaplan–Meier curves with log-rank tests. The optimal threshold of myocardial blood flow on CT–MPI was identified with X-tile software, which is widely used for determining outcome-related cut points in survival analyses (19). Cox proportional hazards models were used to estimate hazard ratios and 95% confidence intervals for baseline characteristics, OCAD, T2DM, and CT–MPI parameters. The proportional hazards assumption was evaluated and verified using Schoenfeld residuals. Statistical significance was defined as a two-tailed *p* < 0.05. All statistical analyses were conducted using R software (version 4.0.1).SPSS (version 29.0; IBM Corp.), and GraphPad Prism (version 10.0; San Diego, CA, USA).

## 3 Results

### 3.1 Study characteristics

Baseline characteristics are shown in Table 1. Sex, age, and other clinical risk factors (hypertension, dyslipidemia, smoking, and BMI) did not differ significantly among the four groups (all *p* > 0.05). The overall difference in symptoms did not reach statistical significance (*p* = 0.088), whereas angina differed significantly among groups (*p* = 0.003). CAD-RADS category and CACS distribution also differed significantly among the four groups (both *p* < 0.001). Glucose and Glycosylated hemoglobin differed among the groups, but showing no significant difference among T2DM (OCAD−) and T2DM (OCAD+) groups. Known diabetes duration was similar between two T2DM groups.

**Table 1:** Baseline characteristics of the study cohort.

Characteristics	Control group		T2DM group		P
	Control (OCAD-) (*n* = 125)	Control (OCAD+) (*n* = 99)	T2DM (OCAD-) (*n* = 100)	T2DM (OCAD+) (*n* = 93)	
Male, *n* (%)	80 (64.0%)	64 (64.6%)	59 (59.0%)	64 (68.8%)	0.564
Age (years)	63 (57–70)	64 (57–72)	64 (56–73)	65 (58–72)	0.498
BMI (kg/m^2^)	24.5 (22.1–26.7)	24.6 (22.5–26.3)	24.1 (21.7–26.9)	23.8 (22.4–25.4)	0.633
Hypertension, *n* (%)	64 (51.2%)	65 (65.6%)	56 (56.4%)	56 (60.2%)	0.138
Dyslipidemia, *n* (%)	34 (27.2%)	25 (25.3%)	35 (34.7%)	28 (30.1%)	0.449
Smoking, *n* (%)	33 (26.4%)	21 (21.2%)	22 (21.8%)	28 (30.1%)	0.420
CAD-RADS category					-
CAD-RADS 0	83	0	58	0	
CAD-RADS 1	2	0	15	0	
CAD-RADS 2	40	0	27	0	
CAD-RADS 3	0	62	0	79	
CAD-RADS 4	0	37	0	14	
CACS					0.069
0	86	47	61	53	
1–100	27	21	26	15	
101–400	9	13	10	12	
＞400	3	18	3	13	
Symptoms, *n* (%)	92 (73.6%)	81 (81.8%)	86 (86.1%)	78 (83.9%)	0.088
Angina, *n* (%)	27 (21.6%)	26 (26.3%)	28 (28.7%)	41 (44.1%)	0.003
Glucose (mmol/L)	5.30 (4.99–5.59)	5.62 (5.21–6.36)	6.66 (5.62–8.11)	6.64 (5.84–8.05)	<0.001
Glycosylated hemoglobin (%)	5.55 (5.30–5.90)	5.9 (5.6–6.4)	6.80 (6.25–8.15)	6.70 (6.32–7.60)	<0.001
Known diabetes duration (years)	-	-	10 (3–14)	9 (4–11)	-

T2DM, type 2 diabetes mellitus; OCAD, obstructive cardiac artery disease; BMI, body mass index; CAD-RADS, coronary artery disease-reporting and data system; CACS, coronary agatston calcium score.

### 3.2 Comparison of CCTA and CT–MPI parameters among the four groups

CCTA revealed the stenosis degree and CACS results of 93 patients with CAD-RADS grades 3–4 and 100 patients with CAD-RADS grades 0–2 in T2DM groups. In the T2DM (OCAD+) group, 79 of 93 patients had CAD-RADS grade 3. Meanwhile, in the T2DM (OCAD−) group, 58 of 100 did not have coronary artery disease (CAD). In our cohorts, there were no patients with CAD-RADS grade 5. 83 of 125 patients in the control (OCAD-) group did not have CAD. 62/99 patients in the control (OCAD+) group had CAD-RADS grade 3. The degrees of coronary artery stenosis in each group are presented in Table 1.

The CT–MPI parameters of the observed groups are listed in Table 2 and Figures 2 and 3. The comparison of CT–MPI parameters among four groups are listed in Supplementary Table 1. All measured parameters exhibited excellent reproducibility both between and within observers (ICC > 0.75, *p* < 0.001). The MBF stepwise reduced from the control (OCAD-), T2DM (OCAD−), control (OCAD+) to the T2DM (OCAD+) group in different segments and global left-ventricular (*p* < 0.05). Compared to controls, both T2DM (OCAD+) and T2DM (OCAD−) patients exhibited impaired MMI, as reflected by reduced MBF and prolonged TTP in the global myocardium and across all three short-axis levels (*p* < 0.05). Furthermore, the T2DM (OCAD+) group had worse MMI than T2DM (OCAD−) and control groups, as evidenced by a more reduced MBF in the global and all three short-axis segments (*p* < 0.05) (Supplementary Table 2).

**Table 2:** CT-MPI parameters among control, T2DM (OCAD-) and T2DM (OCAD+) groups.

Parameters	Control group		T2DM group		*p*
	Control (OCAD-) (*n* = 125)	Control (OCAD+) (*n* = 99)	T2DM (OCAD-) (*n* = 100)	T2DM (OCAD+) (*n* = 93)	
Basel					
MBF	161.9 (140.4–187.7)	141.7 (120.6–157.8)	141.6 (125.4–162.7)	136.4 (109.1–153.6)	<0.001
MBV	17.2 ± 2.4	16.4 (15.2–17.8)	16.7 ± 3.5	15.9 ± 2.9	0.016
PCBV	9.9 (8.4–12.0)	8.7 (7.3–9.8)	10.0 (7.8–11.4)	8.9 (7.1–10.5)	<0.001
FE	95.6 (78.5–115.1)	98.9 ± 23.0	101.2 (83.2–117.2)	93.5 (71.6–107.0)	<0.001
TTP	10.3 (9.5–11.1)	10.8 (10.2–11.8)	11.2 (10.0–12.3)	11.2 (10.0–12.1)	<0.001
Mid-ventricular					
MBF	177.2 (153.3–202.0)	149.6 (130.8–165.8)	155.0 (135.1–172.4)	148.3 (113.2–165.4)	<0.001
MBV	19.3 (17.3–20.5)	16.7 ± 2.6	18.0 (16.4–19.5)	17.2 (15.1–19.3)	<0.001
PCBV	11.4 (9.2–13.0)	9.5 (7.6–11.1)	10.5 (8.8–11.9)	9.9 (7.7–11.8)	0.303
FE	107.7 (85.6–126.4)	103.6 ± 25.9	108.2 (87.5–127.3)	97.9 (77.9–113.5)	<0.001
TTP	10.1 (9.5–10.9)	10.6 (10.0–11.6)	11.0 (9.9–12.1)	11.0 (10.0–11.9)	<0.001
Apical					
MBF	174.0 (149.1–199.6)	148.8 (130.9–168.1)	153.9 (129.5–174.4)	144.9 (109.7–161.9)	<0.001
MBV	19.1 (16.9–20.2)	16.8 ± 2.7	17.9 (16.3–19.4)	16.9 (15.3–19.1)	<0.001
PCBV	10.4 (8.9–12.3)	9.6 ± 3.3	9.9 (8.2–11.8)	9.7 (7.5–11.7)	0.400
FE	106.9 (89.1–131.3)	115.5 ± 28.5	115.8 (94.5–134.5)	98.4 (80.7–114.0)	<0.001
TTP	10.2 (9.6–11.0)	10.6 (9.9–11.8)	11.1 (10.1–12.4)	11.1 (9.9–11.9)	<0.001
Global					
MBF	170.4 (150.0–199.0)	146.8 (127.9–163.3)	151.8 (132.8–172.0)	144.8 (108.7–159.7)	<0.001
MBV	18.5 (16.6–19.9)	16.5 (15.2–18.3)	17.5 (16.0–19.1)	16.6 (14.9–18.7)	<0.001
PCBV	10.5 (8.7–12.1)	9.3 (7.3–10.8)	10.2 (8.2–11.5)	9.5 (7.1–11.6)	0.190
FE	102.7 (85.4–123.5)	105.7 ± 25.9	107.3 (87.4–126.3)	96.9 (79.1–111.0)	<0.001
TTP	10.3 (9.5–11.0)	10.7 (10.0–11.7)	11.1 (10.0–12.3)	11.1 (9.8–11.8)	<0.001

T2DM, Type 2 diabetes mellitus; MBF, myocardial blood flow; MBV, myocardial blood volume; PCBV, perfusion capillary blood volume; FE, flow extraction; TTP, time to peak.

**Figure 2:**
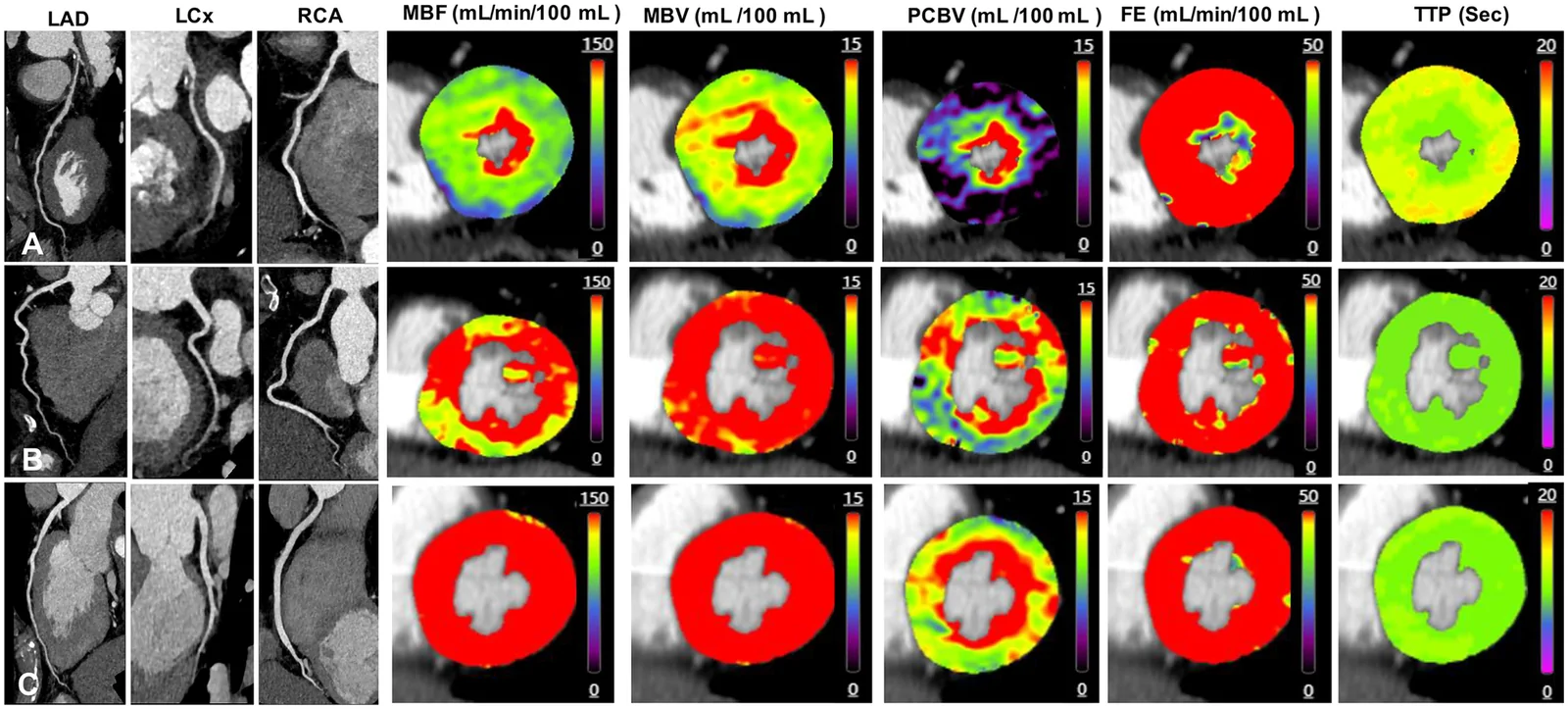
Quantitative analysis of CT-MPI in patients with and without T2DM with and without OCAD. **A**: Representative case of a 56-year-old diabetic male with unstable chest pain. CCTA revealed the atherosclerosis of the OCAD with CAD-RADS 4. The CT-MPI showed that the MBF, MBV and PCBV decreased and TTP increased in all segments, indicating the presence of microvascular ischemia. This patient got an acute myocardial infarction one month after CT-MPI scan. **B**: Representative case of a 64-year-old female patient with diabetes. CCTA revealed normal coronary vasculature. The CT-MPI showed that the MBF and PCBV decreased in the basal inferior segment, indicating the presence of microvascular ischemia. This patient got no MACEs after 32 months follow-up. **C**: Representative case of a 54-year-old female patient without diabetes. CCTA revealed normal coronary vasculature. The CT-MPI showed that the parameters were within the normal range, indicating the absence of microvascular ischemia. LAD, left anterior descending artery; LCx, left circumflex artery; RCA, right coronary artery; MBF, myocardial blood flow; MBV, myocardial blood volume; PCBV, perfusion capillary blood volume; FE, flow extraction; TTP, time to peak.

**Figure 3:**
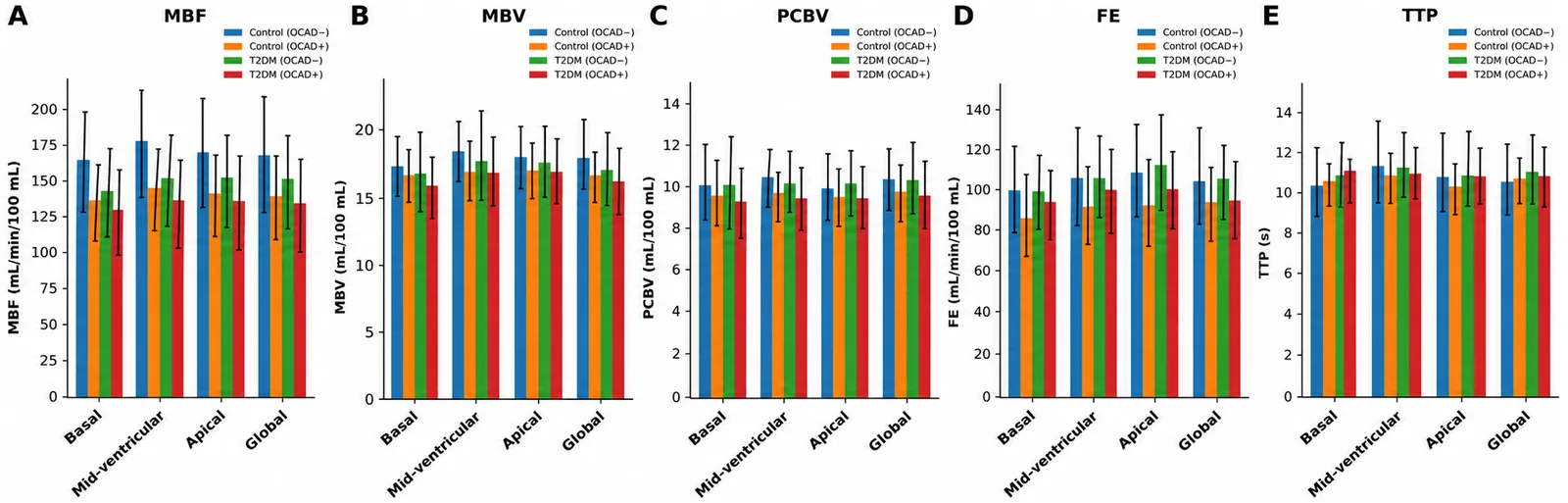
Comparison of CT-MPI parameters at the basal, mid-ventricular, apical, and global left-ventricular levels among control (OCAD-), control (OCAD+), T2DM (OCAD-), and T2DM (OCAD+) groups. **A–E**: MBF, MBV, PCBV, FE, and TTP, respectively. Overall group differences were significant for global MBF, MBV, PCBV, FE, and TTP. MBF, myocardial blood flow; MBV, myocardial blood volume; PCBV, perfusion capillary blood volume; FE, flow extraction; TTP, time to peak; T2DM, type 2 diabetes mellitus; OCAD, obstructive coronary artery disease.

### 3.3 Incremental effect of OCAD on CT–MPI parameters in patients with T2DM

As shown in Table 3, univariate analysis revealed the statistical significance between global MBF with age (r = −0.36, *p* = 0.010), symptoms (r = −10.88, *p* = 0.009), angina (r = −11.36, *p* = 0.002), smoking (r = −9.18, *p* = 0.016), T2DM (r = −14.01, *p* < 0.001), and OCAD (r = −19.06, *p* < 0.001); sex was also associated with global MBF (r = 12.41, *p* < 0.001). With CAD-RADS 0 as the reference category, when CAD-RADS was entered into the regression model alone, CAD-RADS 1, 3, and 4 were significantly associated with lower global MBF, whereas the association for CAD-RADS 2 was not statistically significant. Multivariate linear regression analyses were conducted to determine the additive influence of OCAD on MMI in patients with T2DM (Table 4). After adjusting for age, sex and angina, OCAD and T2DM were individually included in the model, showing that each was independently associated with global MBF (OCAD: *p* < 0.05, R^2^ = 0.154; T2DM: *β* = −11.96, *p* < 0.001, R^2^ = 0.101). With adjustment for the above covariates, the combined model including OCAD and T2DM demonstrated a significant correlation between OCAD and global MBF in the context of T2DM (*p* < 0.05, R^2^ = 0.176). Notably, the relationship between CAD-RADS category and global MBF does not necessarily demonstrate a strict grade-dependent pattern.

**Table 3:** Univariable linear regression analyses correlations of T2DM and OCAD with global CT-MPI parameters.

Global-MBF	Univariable		
	r	95% CI	*p*
Sex	12.41	5.78 to 19.04	<0.001
Age	−0.36	−0.63 to −0.09	0.010
BMI	−0.56	−1.48 to 0.36	0.232
Symptoms	−10.88	−19.02 to −2.73	0.009
Angina	−11.36	−18.39 to −4.34	0.002
Hypertension	−5.29	−11.83 to 1.26	0.113
Dyslipidemia	−1.61	−8.72 to 5.49	0.656
Smoking	−9.18	−16.61 to −1.75	0.016
CAD-RADS category			
1	−24.56	−40.70 to −8.42	0.003
2	−1.55	−10.87 to 7.78	0.745
3	−20.78	−28.27 to −13.29	<0.001
4	−23.04	−33.31 to −12.76	<0.001
CACS	−0.001	−0.01 to 0.01	0.918
T2DM	−14.01	−20.35 to −7.66	<0.001

MBF, myocardial blood flow; BMI, body mass index; CAD-RADS, coronary artery disease-reporting and data system; CACS, coronary agatston calcium score; T2DM, type 2 diabetes mellitus.

**Table 4:** Associations between global CT-MPI parameters, and CAD-RADS, T2DM as well as other clinical factors.

Global-MBF	Multivariable			
	*β*	95% CI	*p*	R2
Model 1: T2DM + Sex + Age + Angina				0.101
T2DM	−11.96	−18.25 to −5.66	<0.001	
Model 2: CAD-RADS + Sex + Age + Angina				0.154
CAD-RADS 1 vs. 0	−28.59	−44.45 to −12.73	<0.001	
CAD-RADS 2 vs. 0	−0.41	−9.50 to 8.68	0.930	
CAD-RADS 3 vs. 0	−17.86	−25.31 to −10.40	<0.001	
CAD-RADS 4 vs. 0	−19.76	−29.84 to −9.68	<0.001	
Model 3: T2DM + CAD-RADS + Sex + Age + Angina				0.176
T2DM	−10.52	−16.81 to −4.23	0.001	
CAD-RADS 1 vs. 0	−22.86	−38.90 to −6.81	0.005	
CAD-RADS 2 vs. 0	−0.59	−9.58 to 8.39	0.897	
CAD-RADS 3 vs. 0	−16.82	−24.21 to −9.44	<0.001	
CAD-RADS 4 vs. 0	−21.48	−31.50 to −11.47	<0.001	

MBF, myocardial blood flow; T2DM, type 2 diabetes mellitus; CAD-RADS, coronary artery disease-reporting and data system.

### 3.4 Outcomes

Across a median follow-up of 24 months (IQR, 11–42), 19 MACEs (6.9%) were observed: 3 deaths, 10 myocardial or cerebral infarctions, 4 severe arrhythmias, and 2 hospitalization for unstable angina. Two patients died and one patient experienced severe arrhythmia in the T2DM (OCAD−) group. Five patient had MACEs in the control group, with one patient in the control (OCAD-) had unstable angina requiring hospitalization, and 2 myocardial infarctions, 1 severe arrhythmias, and 1 hospitalization for unstable angina in the control (OCAD-) group. Notably, the incidence of MACEs was significantly higher in the T2DM (OCAD+) group (11/64, 17.2%) than in the T2DM (OCAD−) group (3/49, 6.1%). The cumulative hazard curves constructed using Kaplan–Meier survival analysis are presented in Figure 4. The global MBF was categorized as high or low risk based on the results of X-tile software depending on whether it is less than 110. Higher event rates and early separation of the event curves were observed in patients with high-risk global MBF and those in the T2DM (OCAD+) group (log-rank test, both *p* < 0.01). As shown in Table 5, Figure 4B and Supplementary Figure 1, the univariate Cox regression analysis revealed that CAD-RADS (*p* = 0.010) and global MBF (*p* < 0.001) were significantly associated with MACEs in patients with T2DM. In the multivariate stepwise Cox regression analysis, global MBF was independently associated with MACEs in patients with T2DM (*p* < 0.05).

**Figure 4:**
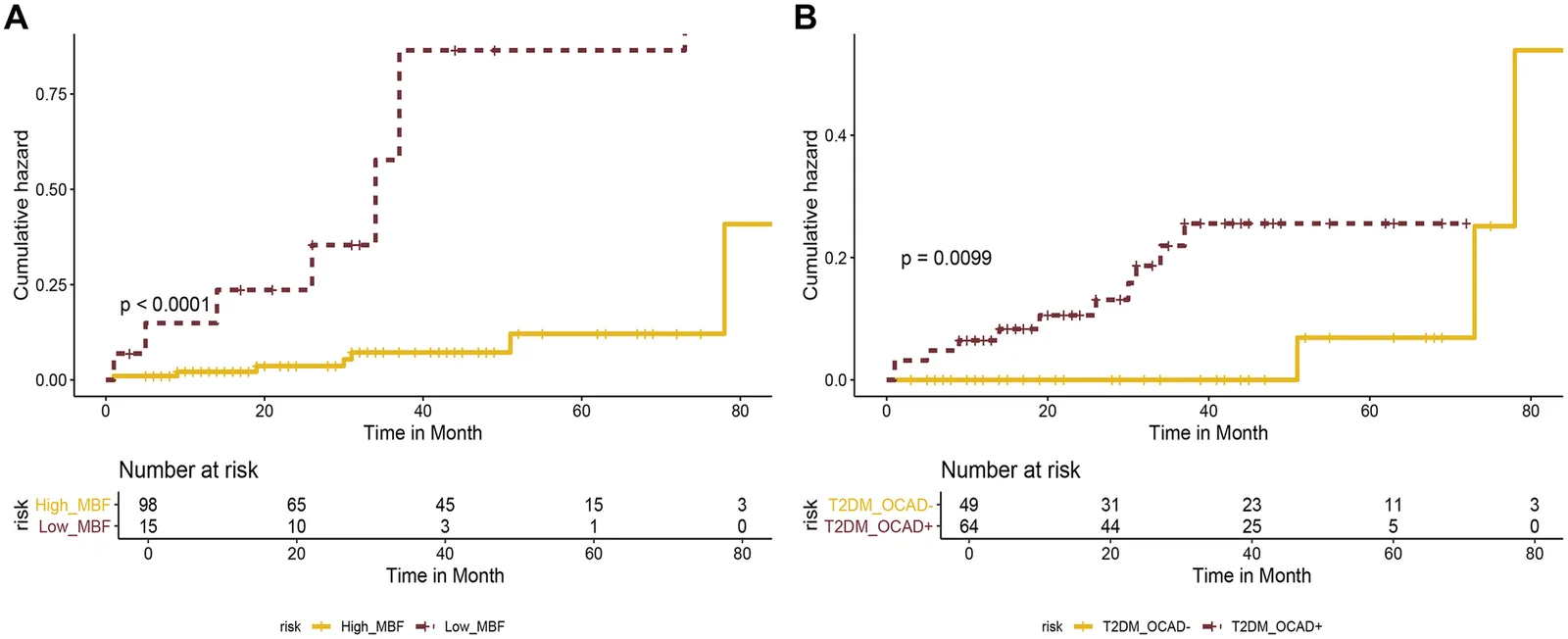
Kaplan–Meier estimates of cumulative hazard for MACEs in patients with T2DM. (A) Cumulative hazard curves stratified by MBF status (high MBF vs. low MBF). (B) Cumulative hazard curves stratified by the presence of OCAD (OCAD− vs. OCAD+). MACEs, major adverse cardiovascular events; T2DM, type 2 diabetes mellitus; MBF, myocardial blood flow; OCAD, obstructive coronary artery disease.

**Table 5:** Univariable predictors for MACEs of the diabetes patients.

Predictors	All (*n* = 113)	No event (*n* = 99)	Event (*n* = 14)	HR (95%CI)	*p*
Male	77 (68.14%)	66 (66.67%)	11 (78.57%)	1.08 (0.29–4.03)	0.903
Age	66.5 ± 11.4	66.6 ± 11.1	65.5 ± 13.8	0.96 (0.92–1.01)	0.138
BMI	23.7 (21.8–25.4)	23.7 (21.8–25.4)	23.5 (22.0–24.8)	1.01 (0.88–1.16)	0.892
Symptoms	94 (83.19%)	83 (83.84%)	11 (78.57%)	0.91 (0.25–3.26)	0.881
Angina	37 (32.74%)	34 (34.34%)	3 (21.43%)	0.53 (0.15–1.93)	0.331
Hypertension	68 (60.18%)	59 (59.60%)	9 (64.29%)	1.18 (0.40–3.54)	0.766
Dyslipidemia	29 (25.66%)	25 (25.25%)	4 (28.57%)	1.08 (0.33–3.53)	0.905
Smoking	30 (26.55%)	26 (26.26%)	4 (28.57%)	1.19 (0.36–3.95)	0.777
CACS	0.0 (0.0–34.9)	0.00 (0.00–26.6)	6.5 (0.00–118.1)	1.00 (1.00–1.00)	0.718
CAD-RADS	64 (56.64%)	53 (53.54%)	11 (78.57%)	9.40 (1.20; 73.8)	0.010
Glucose	6.61 (5.59–8.05)	6.62 (5.60–8.00)	6.46 (5.35–8.86)	2.00 (0.78–1.28)	0.984
Glycosylated hemoglobin	6.65 (6.20–7.85)	6.70 (6.20–7.80)	6.55 (6.08–7.97)	0.99 (0.84–1.18)	0.945
Global-MBF	15 (13.27%)	8 (8.08%)	7 (50.00%)	9.41 (3.11;28.4)	<0.001

BMI, body mass index; CACS, coronary agatston calcium score; CAD-RADS, coronary artery disease-reporting and data system; MBF, myocardial blood flow.

## 4 Discussion

The principal findings of this study can be summarized as follows: 1) T2DM was associated with MMI, and OCAD may exacerbate MMI in patients with T2DM; 2) the CT–MPI parameters were the independent predictors of MACEs, which indicated the prognostic value in patients with T2DM.

MMI has been increasingly recognized as an essential component of DM (20, 21). MMI arises from reduced angiogenesis due to decreased myocardial vascular endothelial growth factor (VEGF) and receptor expression, in conjunction with endothelial dysfunction and elevated microvascular stiffness (22). The diagnostic value of cardiac magnetic resonance was clearly proven for cardiac function differences between the T2DM and control groups, including association with diabetic complications (23) and prognosis of MACEs (7). Meanwhile, a previous study demonstrated reduced myocardial perfusion parameters in patients with diabetes using dynamic CT–MPI (24). In the present four-group analysis, global MBF was significantly lower in both the control (OCAD+) and T2DM groups than in the control (OCAD-) group, and the lowest value was observed in T2DM (OCAD+) patients. These findings emphasize that impaired myocardial perfusion in T2DM should be interpreted in the context of concomitant epicardial coronary disease rather than attributed to diabetes alone.

A majority of patients with T2DM have OCAD in the real world. In the current study, the evaluation of the T2DM (OCAD−) group was deemed insufficient. The pathophysiological mechanisms of OCAD on MMI remain unclear. Several potential mechanisms including constriction of periarteriolar and arteriolar vessels have been proposed, as well as inadequate subepicardial periarteriolar dilation in response to increased myocardial oxygen demand (25). Wang et al. reported that the T2DM (OCAD+) group exhibited a greater reduction in CMR parameters than T2DM (OCAD−) and control groups (26). However, CMR has limitations in assessing microvascular ischemia in diabetes, as it provides only functional evaluation and cannot identify OCAD (6). CT–MPI combined with CCTA offers a comprehensive approach, allowing simultaneous assessment of both OCAD and MMI in T2DM (27), highlighting the potential for simultaneous diagnosis of OCAD and MMI in patients with T2DM. After ruling out the hemodynamic significance of OCAD, we investigated the difference in CT–MPI parameters between T2DM (OCAD−) and (OCAD+) groups. The reduction in MBF indicated that OCAD exacerbates MMI in patients with T2DM. The relationship between epicardial coronary arteries and coronary microcirculation was assessed using univariate and multivariate linear regression analyses. The findings further demonstrated that OCAD independently predicts MMI in patients with T2DM. Early and appropriate medical management may be warranted to mitigate MMI and potentially reduce the risk of microcirculation-related complications. Notably, our findings suggested that the relationship between CAD-RADS category and global MBF may not be strictly linear or monotonic. CAD-RADS primarily reflects the anatomical severity of coronary artery stenosis, whereas global MBF reflects the integrated effects of not only epicardial coronary artery disease but also diffuse coronary atherosclerosis, coronary microvascular dysfunction, and diabetes-related microcirculatory impairment.

MMI has been recognized as an adverse prognostic marker in individuals with T2DM (8). Multiple studies have demonstrated that various imaging- or perfusion-based assessment methods possess meaningful predictive value for MACEs in this population (8, 28, 29). Prior evidence also indicates that patients with diabetes exhibit reduced MBF, independent of traditional cardiovascular risk factors and irrespective of the presence of obstructive coronary disease (30). In addition, Xue et al. showed that CT–MPI offers incremental prognostic information beyond clinical variables alone or in combination with CCTA characteristics for predicting MACEs in both diabetic and non-diabetic cohort (31). These findings collectively support the prognostic relevance of CT–MPI–derived parameters in T2DM. In our study, patients with T2DM and concomitant OCAD experienced a markedly higher incidence of MACEs compared with those without OCAD, suggesting that epicardial obstruction may exacerbate MMI and further worsen clinical outcomes. Moreover, global MBF emerged as an independent predictor of MACEs in the T2DM population. This observation aligns with prior research demonstrating the robust diagnostic capability of MBF to differentiate flow-limiting from non–flow-limiting coronary stenosis, achieving high sensitivity and specificity (84.3% and 97.7%, respectively) (32), thus reinforcing the clinical value of MBF evaluation.

Despite these valuable findings, the present study had several limitations. First, current imaging techniques cannot distinguish the hemodynamic significance of OCAD and MMI. In our study, patients with both vascular stenosis and myocardial ischemia at the corresponding territory were excluded, although those with concomitant MMI were not excluded. Such exclusion introduced a certain degree of sample bias. This study did not include patients with CAD-RADS grade 5 due to the hemodynamic significance of OCAD. Therefore, such patients still need to be investigated in future studies. Second, the influence of left ventricular remodeling measured by CT–MPI was not evaluated in this study. Third, imaging information were not available on cardiac interstitial fibrosis. Thus, future investigation is warranted to confirm cardiac interstitial fibrosis in patients with T2DM. Finally, A retrospective, single-center observational study was conducted involving patients who received third-generation dual-source CT. Validation and generalization of these findings would benefit from prospective and multicenter studies.

## 5 Conclusion

In conclusion, CT–MPI combined with CCTA showed an increased incidence of MMI in diabetes patients with OCAD than in those without OCAD. Moreover, MBF is strongly related with the prognosis in T2DM patients.

## Data Availability

The original contributions presented in the study are included in the article/Supplementary Material, further inquiries can be directed to the corresponding author/s.
